# Counselor Efficiency at Providing Feedback in a Technology-Based Behavioral Weight Loss Intervention: Longitudinal Analysis

**DOI:** 10.2196/23974

**Published:** 2021-05-05

**Authors:** Margaret C Fahey, Robert C Klesges, Mehmet Kocak, Leslie A Gladney, Gerald W Talcott, Rebecca A Krukowski

**Affiliations:** 1 Psychology Department The University of Memphis Memphis, TN United States; 2 School of Medicine University of Virginia Charlottesville, VA United States; 3 Department of Preventive Medicine College of Medicine University of Tennessee Health Science Center Memphis, TN United States

**Keywords:** technology-based intervention, counselor communication, counselor feedback, counselor, weight loss, lifestyle, wellness

## Abstract

**Background:**

Feedback for participants’ self-monitoring is a crucial and costly component of technology-based weight loss interventions. Detailed examination of interventionist time when reviewing and providing feedback for online self-monitoring data is lacking.

**Objective:**

The aim of this study was to longitudinally examine the time counselors spent providing feedback on participant self-monitoring data (ie, diet, physical activity, weight) in a 12-month technology-based weight loss intervention. We hypothesized that counselors would compose feedback for participants more quickly over time.

**Methods:**

The time the lay counselors (N=10) spent reviewing self-monitoring records and providing feedback to participants via email was longitudinally examined for all counselors across the three years of study implementation. Descriptives were observed for counselor feedback duration across counselors by 12 annual quarters (ie, 3-month periods). Differences in overall duration times by each consecutive annual quarter were analyzed using Wilcoxon-Mann-Whitney tests.

**Results:**

There was a decrease in counselor feedback duration from the first to second quarter (mean 53 to 46 minutes; *P*<.001), and from the second to third (mean 46 to 30 minutes; *P*<.001). A trend suggested a decrease from the third to fourth quarter (mean 30 to 26 minutes; *P*=.053), but no changes were found in subsequent quarters. Consistent with the hypothesis, counselors may be increasing their efficiency in providing feedback; across 12 months, counselors spent less time reviewing participant self-monitoring and composing feedback (decreasing from mean 53 to 26 minutes).

**Conclusions:**

Counselors used increasingly less time to review online self-monitoring data and compose feedback after the initial 9 months of study implementation. Results inform counselor costs for future technology-based behavioral weight loss interventions. For example, regardless of increasing counselor efficiency, 25-30 minutes per feedback message is a high cost for interventions. One possibility for reducing costs would be generating computer-automated feedback.

**Trial Registration:**

ClinicalTrials.gov NCT02063178; https://clinicaltrials.gov/ct2/show/NCT02063178

## Introduction

Consistent weight and dietary self-monitoring are key elements for successful weight loss in both in-person [1] and technology-based programs [2], and using technology for self-monitoring (eg, apps, smart scales) can increase self-monitoring adherence [3-5]. Personalized self-monitoring feedback on the frequency of weight, dietary, and exercise monitoring; reinforcing comments about weight loss behaviors; and presentation of behavior change possibilities are core elements of behavioral weight loss interventions [6-8]. Feedback on self-monitoring data appears to be a crucial component of these interventions since it is associated with greater self-monitoring engagement as well as greater weight loss in interventions [9-12]. In recent years, technology-based communication (eg, email) has been increasingly used for counselor’s feedback [9], particularly since participants are now able to self-monitor food intake, weight, and physical activity online using either researcher-developed or commercial websites/apps [13] rather than using paper and pencil diaries.

In studies that have evaluated the cost-effectiveness of behavioral weight loss interventions [14-25], interventionist compensation emerges in the available studies as one of the costliest components [21,24,25], including time for conducting the sessions and for providing feedback (ie, review of self-monitoring data, composing feedback). Interventionist costs, however, are often bundled in these analyses [21,24,25]; that is, combining time required for the sessions together with time required for providing feedback as well as other intervention tasks. The amount of time for sessions is often quite rigid (eg, 60-90 minutes for group sessions; 20-30 minutes for individual sessions), with standard outlines of material to cover, but little is known about the time associated with providing self-monitoring feedback.

One study examined costs of providing email feedback for online self-monitoring data, based on retrospective self-reported estimates of counselor time in a “typical week” and “after substantial implementation experience,” but bundled all intervention costs together (ie, session preparation, conducting the group session, review of self-monitoring journals, periodic contact of participants who might miss sessions or who have questions, posting on the bulletin board, record keeping, technical work by the webmaster) [21,24]. To our knowledge, only one behavioral intervention specifically reported isolated counselor feedback time, using an average weekly estimate for the entire intervention, but it is not clear whether this information was collected contemporaneously or retrospectively [26]. Thus, previous information is limited to averages, and it is not clear that real-time data collection of each feedback message composed has been examined. Further, because counselor efficiency might increase over time, it will be important to examine the potential impact of implementation experience.

Thus, the purpose of this study was to longitudinally examine the time required to review self-monitoring data and compose feedback among newly trained counselors for participants engaged in a 12-month behavioral weight loss intervention. These data will be important since a detailed examination of interventionist time when reviewing and providing feedback for online self-monitoring data can inform the cost-effectiveness of technology-based programs (including the time for new counselors to “peak” in efficiency) and serve as a baseline for comparison if strategies are implemented for increasing efficiency. This study examined time spent on counselor self-monitoring feedback across the three years that the weight loss intervention was implemented. We hypothesize that counselors will deliver feedback on participant diet, physical activity, and weight self-monitoring more quickly over time.

## Methods

### Participants

Individuals receiving self-monitoring feedback in the behavioral weight loss intervention were active duty military personnel stationed at Lackland Air Force Base in San Antonio, Texas, enrolled in the Fit Blue weight loss study (2014-2017) [27,28]. Recruitment used posters and bulletins, on-base presentations, advertisements, and word of mouth. Those interested were phone screened by study staff to assess eligibility (ie, >1 year left on base, >18 years of age, BMI >25.0 kg/m^2^, health care provider clearance, computer/email access). At baseline, 248 participants were randomized to either a counselor-initiated or self-paced 12-month intervention condition. Conditions varied in intensity and self-initiation required but were similar in intervention goals.

### Self-monitoring Components

#### Overview

Participants were asked to self-monitor food intake, physical activity, and weight daily. To record food intake and physical activity, participants used the Lose It! app or website and permitted their counselor to access this information. To monitor weight, participants used the BodyTrace e-scale provided to them at baseline, which uploaded to a secure personalized website. In the counselor-initiated condition, counselors provided feedback on dietary, physical activity, and weight self-monitoring at the same frequency as telephone sessions (ie, weekly for 4 months, then biweekly for 4 months, then monthly for 4 months) via email (28 total). The self-paced condition was provided feedback via email when requested (up to 28), although in practice this feedback was rarely requested in the self-paced condition [28]. The time estimates presented in the analyses are based on 2670 emails (Table 1). The protocol was approved by the Institutional Review Board of the Wilford Hall Ambulatory Surgical Center in San Antonio, Texas, and acknowledged by the Institutional Review Board at the University of Tennessee Health Science Center.

**Table 1:** Change in feedback durations across counselors.

Quarters from first review	Feedback duration across counselors (minutes)				
	Number of feedback emails	Quartile 1	Median	Mean (SD)	Quartile 3
1	335	30	30	53 (54)	60
2	380	20	30	46 (47)	45
3	324	20	30	30 (25)	30
4	286	15	25	26 (19)	30
5	317	20	30	26 (12)	30
6	309	17	25	27 (15)	30
7	249	20	30	28 (14)	30
8	231	25	30	27 (7)	30
≥9	239	25	30	28 (12)	30

#### Counselor Characteristics and Training

Counselors (N=10) held bachelor’s or master’s degrees (ie, social work, counseling/psychology, child and family development, nursing, justice administration); however, they were considered lay interventionists since no prior counseling or research experience was required. Counselors were hired based on their interest in providing behavioral interventions and in research, and they were either retired from the military or familiar with military culture. All counselors were new to providing self-monitoring feedback in a weight loss intervention. Counselors attended a week-long training on the study protocol, behavioral weight management principles, feedback, and motivational interviewing. Counselors were taught to construct emails as “feedback sandwiches,” with reinforcement of behaviors sandwiched around identification of potential areas of behavior change, consistent with guidance from other studies [29]. Counselors submitted practice emails that were discussed among counselors led by the principal investigator (RAK). Two counselors joined the team midway through the study timeline and were similarly trained. All counselors participated in ongoing biweekly 1-hour supervision and 1-hour motivational interviewing training and received roughly quarterly retraining on providing self-monitoring feedback to maintain and improve this skill.

#### Documentation of Self-monitoring Feedback Duration

Counselors contemporaneously logged the time it took them to review online self-monitoring data and construct each personalized email in the electronic study database.

### Data Analysis

All feedback duration times were included in analyses regardless of condition. Descriptives (ie, median, mean, SD, first quartile, third quartile, quartile range) were observed for feedback duration across counselors by 12 annual quarters (ie, 3-month periods). For counselors who joined the team later (N=2), the first quarter they provided feedback was compiled with the first quarter of feedback from the original counselors. Differences in overall duration times by each consecutive annual quarter were analyzed using the Wilcoxon-Mann-Whitney test.

## Results

**Figure 1:**
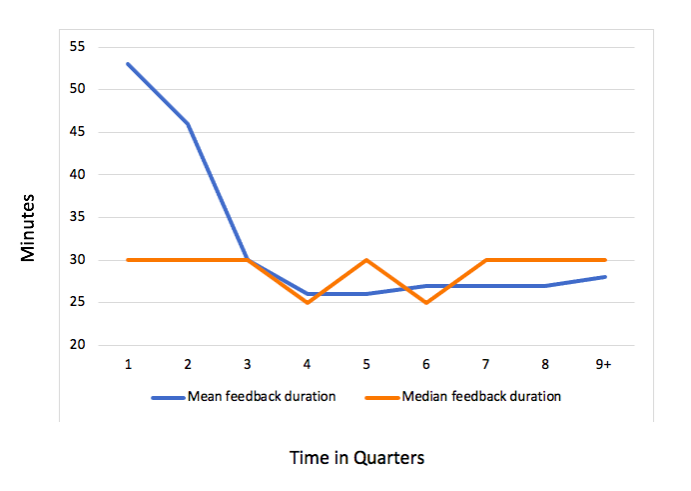
Feedback duration across intervention quarters. Counselor efficiency increases over time.

Across all counselors, there was a significant decrease in overall duration to review self-monitoring data and compose feedback messages from the first to second quarter (*P*<.001; IQR 30-60 minutes versus 20-45 minutes; mean 53, SD 56 versus mean 46, SD 47; Table 1). There was a significant decrease in overall duration from the second to third quarter (*P*<.001; IQR 20-45 minutes versus 20-30 minutes; mean 46, SD 47 versus mean 30, SD 25). A nonsignificant trend suggested a decrease in duration from the third to fourth quarter (*P*=.053; IQR 20-30 minutes versus 15-30 minutes; mean 30, SD 25 versus mean 26, SD 19). There was no significant change in duration between later quarters (Table 1). Median time ranged from 25 to 30 minutes (Table 1). Median and mean feedback durations across all quarters are presented in Figure 1.

## Discussion

### Principal Findings

Counselors needed increasingly less time to review online self-monitoring data and compose personalized feedback to participants over the first 9 months the behavioral weight loss intervention was implemented, with a nonsignificant trend suggesting increased efficiency for the next 3 months as well. Thus, after 12 months, the mean amount of time spent in reviewing self-monitoring data and composing feedback decreased from 53 to 26 minutes. When examining median times, there was less variation across all quarters (ie, 25-30 minutes); however, a decrease in IQR in the first three quarters was notable. This narrowing time range indicated that counselors composed these feedback messages more consistently near the median (ie, 30 minutes) over time.

Although the standard deviation decreased, it remained high, likely due to variability across individual counselors and participant characteristics. Some participants might have logged similar data to previous weeks, or only logged one day, requiring shorter feedback messages. In response to these self-monitoring situations, counselors might write, “You continued to meet your calorie and fat goals and continue to make regular choices of fruits, vegetables, whole grains, and low- or no-calorie beverages” or “You met your calorie and fat goals on the one day that you were able to log. What were the barriers for logging on the other days?” Additionally, perhaps some counselors were quicker at crafting feedback than others.

Mean time was 32 minutes per feedback message, which is higher than the 10-15 minutes found by Hunter et al [26], during which counselors similarly provided feedback on food intake, exercise, and weight. However, only time spent providing feedback was reported, which—unlike the current study—did not include time reviewing self-monitoring data [26]. Further, it is unclear if the counselors in this previous research logged feedback duration contemporaneously or retrospectively [26].

The decrease in time for feedback messages might be influenced by multiple factors. Perhaps counselors became more efficient at reviewing self-monitoring data and constructing feedback with experience and additional training. Another possibility is that counselors provided less detailed feedback and became “sloppier” over time. However, this is less likely given that the overall intervention results indicated participants experienced significant weight loss at 4-month and 12-month outcomes [28], and periodic retraining on self-monitoring feedback was conducted to increase the likelihood of maintaining good quality feedback. Further, participants might have needed increasingly less feedback about their behaviors over the 12 months that they participated in the intervention. However, our study compared time periods specific to counselor experience, which included the three years that the 12-month intervention was implemented. Thus, individual participants cycled throughout the three years that our results were analyzed and overlapped with participants at other stages of intervention. Nonetheless, future research should rate the quality of feedback over time alongside the time required to compose it.

Regardless of increasing counselor efficiency, 25-30 minutes per feedback message is a high cost for interventions, even cost-effective interventions such as this one [24]. In order to improve dissemination of behavioral weight management programs to all who are eligible and interested, particularly in settings (eg, primary care) without individuals who may have the time, training, and supervising experience to provide self-monitoring feedback, it may be beneficial to develop strategies for decreasing the amount of time required for crafting each message. Some possibilities for reducing the costs of individualized counselor feedback would be generating computer-automated feedback or facilitating peer-group interaction to help promote self-monitoring behaviors [30]. Although computer-automated feedback was comparable to counselor feedback in the short term in previous research, automated feedback was less effective for long-term weight loss [12]. Given limited research on computer-generated feedback [12,31,32] and the common use of counselor-generated feedback [9-12], it is clearly still important to understand in detail the time costs associated with counselor feedback. These details can inform future program budgets, especially since counselor compensation is one of the largest costs [21,24,25]. However, since computer-tailored feedback might be more regularly incorporated into future interventions, a better understanding of the efficiency of counselors in crafting feedback may be beneficial to compare these different modalities.

There are several limitations to consider. Counselors self-reported time spent reviewing self-monitoring data and constructing feedback, which may be less accurate compared to objective measurements. However, this level of detail is much greater than previous studies. In addition, findings are based on lay counselors and may differ from other weight loss professionals (eg, dietitians). Finally, future studies might examine a consortium of behavioral weight management studies in order to have a larger sample size of interventionists in analyses that examine these questions.

### Conclusion

Current findings suggest that counselors, across the initial 9 months of a behavioral weight loss intervention, become quicker at reviewing participant self-monitoring data and composing individualized feedback. Although there was individual variability, findings indicate that after 9-12 months of experience, counselors composed self-monitoring feedback more consistently in about 30 minutes. Despite indications of increased counselor efficiency, time per feedback message was only reduced to 25-30 minutes. Weight loss programs might consider testing computer-automated feedback with human tailoring to reduce counselor time.
